# Using GIS in Ecological Management: Green Assessment of the Impacts of Petroleum Activities in the State of Texas

**DOI:** 10.3390/ijerph7052101

**Published:** 2010-05-04

**Authors:** Edmund Merem, Bennetta Robinson, Joan M. Wesley, Sudha Yerramilli, Yaw A. Twumasi

**Affiliations:** 1 Department of Urban and Regional Planning, Jackson State University, 3825 Ridgewood Road, PO Box 23, Jackson, MS 39211, USA; E-Mails: bennetta.robinson@students.jsums.edu (B.R.); joan.m.wesley@jsums.edu (J.W.); ksudhabapu@yahoo.com (S.Y.); 2 Department of Advanced Technologies, School of Agriculture and Applied Sciences, 1000 ASU Drive, Alcorn State University, MS 39096-7500, USA; E-Mail: yaw@alcorn.edu

**Keywords:** GIS, oil and gas, ecological assessment, ecosystem decline, management

## Abstract

Geo-information technologies are valuable tools for ecological assessment in stressed environments. Visualizing natural features prone to disasters from the oil sector spatially not only helps in focusing the scope of environmental management with records of changes in affected areas, but it also furnishes information on the pace at which resource extraction affects nature. Notwithstanding the recourse to ecosystem protection, geo-spatial analysis of the impacts remains sketchy. This paper uses GIS and descriptive statistics to assess the ecological impacts of petroleum extraction activities in Texas. While the focus ranges from issues to mitigation strategies, the results point to growth in indicators of ecosystem decline.

## 1. Introduction

Every facet of energy exploration, recovery, storage processing, and distribution carries some risks associated with environmental impacts. But sometimes these risks are often difficult to assess and costly to anticipate. By developing credible scientific and technological information to characterize those risks, and sharing that data with government regulators and industry operators geo-spatial information technologies such as GIS can minimize and address the problems [1–7]. In the past years, widespread environmental concerns emanating from oil and gas operations prompted the formulation of new regulations across the United States. While these sets of laws laid the structure for much of the environmental mitigation measures adopted by industry, compliance costs have been rising, thereby making things more complicated. In the fiscal year 1996, the petroleum industry, together with refining, spent heavily on nature protection—nearly the same as it paid in the exploration of fresh supplies—with a price tag of $8.2 billion dollars [8]. Some of these issues could have been anticipated in advance and periodically tracked to aid decision making had geospatial technologies been integrated in the policy framework.

In the study area of Texas, where 700,000 to 1 million oil and gas wells were drilled, abandonment and well leakages have emerged as a common occurrence [9]. In 1992, when the state had about 88,000 abandoned oil wells, each of these wells was plugged at a cost of $25,000. Out of these numbers, close to 24,449 abandoned wells contravened the plugging rules set by Texas Railroad Commission. The other leading areas of concern remain problems of oil spills, and atmospheric and water contamination. Minute spills occur with some regularity; however, the large one is time and again the concern. Of major concern are the potential impacts on tourism, air and water. At the same time, soil pollution, mainly from oil refineries and petrochemical operations, also creates additional problem [10]. Accordingly, the issue of pollution is now starting to attract serious attention in North Texas and it may be on the rise. Given the gravity of the impacts, most scholars in the area say it is worth studying to determine the scope of the problem. The level of ground level ozone from hydrocarbons is also on the rise. A study of the Texas Commission on Environmental Quality in 2006 indicated that storage tanks solely accounted for about 38 tons of volatile organic compounds which are equivalent to 7 to 8 percent of the volatile compounds in North Texas airshed. Those chemicals constitute the key elements in ground level ozone, the region’s major pollution problem [11].

Accordingly, geospatial technologies are valuable tools for ecological assessment in stressed environments. Visualization of spatial relationships in these instances between natural features and landscapes prone to ecological disaster associated with oil and gas not only helps in focusing the scope of environmental management analysis with records of changes in affected area, but also it can furnish information on the pace at which resource extraction activities affect the natural environment. With ecosystem protection around oil and gas operations now, key aspects of management in the sector, very little effort has been made by managers to capture the impacts of petroleum activities and trends spatially in Texas. For more information on related studies in other areas see Merem and Twumasi in 2006 [1,2].

With management practices focused solely on production at the expense of conservation and environmental quality, there continues to be widespread concerns on rising costs, scarcity and ecosystem erosion [11–13]. Accordingly, efficient management in the context of hydrocarbon exploration in the state requires commitment towards monitoring of degraded areas using geospatial information systems. Over the past years, GIS and GPS have been used in detecting and mapping the distribution of environmental dis-benefits such as harmful plant varieties [14]. GIS has long been used by researchers as a tool to manage, store, analyze, and display spatial data [15–18]. They provide opportunities for assessing location and the likelihood of damages within an area and the surrounding ecosystem. Assessing the fate of the ecosystem in this setting is a vital contribution to management efforts and the promotion of environmental health strategies needed in oil producing communities of the study area.

Previous studies in sub-Saharan Africa show that GIS and remote sensing offer governments and enterprises a solution for monitoring the carrying capacity of fragile ecosystems impacted by oil and gas activities in areas such as the Niger River Delta. In that work, GIS technology as the authors show fulfills a useful purpose in mapping and inventorying of emission and other related ecological data [1,2]. Geospatial technology also helped quicken the spatial display of the factors, patterns, and environmental effects of oil and gas activities and their implications for global climate change in a region. Integrated data analysis using remotely sensed satellite imagery and GIS modeling facilitated the analysis of the spatial diffusion of CO_2_ emission and the potential environmental change involving forest cover and hydrological changes occurring in the Niger Delta environment across time. These studies show the capacity of GIS to provide valuable information about natural resources, environmental change and basis for sustainable planning.

This paper uses GIS methodology to analyze pressures mounted on the environment in the oil sector of selected counties in the state of Texas for efficient management of the environment. Emphasis is on the issues, factors, management efforts, and future strategies for mitigation. The aims of the paper center on the need to make a contribution to the literature and to design a decision support tool to assist natural resource managers. Other objectives focus on the design of novel geo-spatial methods for analyzing degradation in the oil producing areas of Texas, the need to analyze environmental health issues of pollution with the latest advances in geospatial technologies, and the state of ecosystem health with respect to Texas. The paper is divided into five sections. The first section provides the introduction of the research beginning with a profile of the study area, the issues and benefits; while, the second portion describes the materials and methods. Section three presents the results of data analysis on various themes from temporal profile to geospatial analysis of the impacts of oil and gas activities. In section four, the paper discusses several findings from the research and offers recommendations to address the problems. The fifth and final section highlights the conclusions of the research.

**Figure 1.**
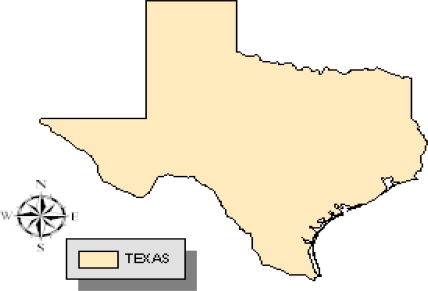
Study Area.

**Figure 2.**
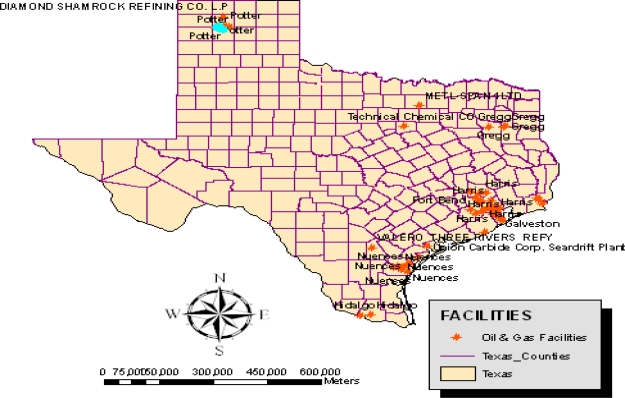
Oil and Gas Facilities.

**Figure 3.**
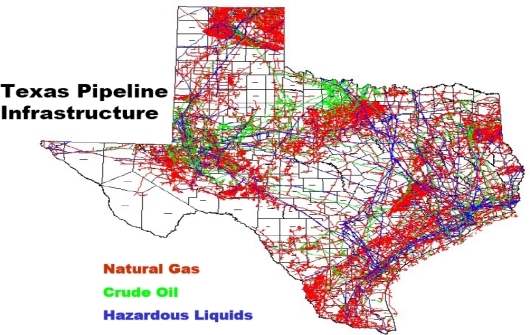
The Spatial Distribution of Pipeline Infrastructure, Source: Carillo, V. (2006) [21].

The study area consists of the oil producing districts of Texas. The state is located very close to the Gulf of Mexico (Figures 1 and 2). With a population of nearly 23 million inhabitants during the last five years, the number of people in the state rose to 24.3 million in 2008. Petroleum infrastructure remains fairly widespread in the area, with a huge network of pipelines and storage facilities (Figure 3). Now, an extensive system of interstate natural gas pipelines run from Texas, serving consumer depots from seaboard to seaboard. The state’s huge natural gas demand serves the industrial and electric power sectors, which jointly account for over four-fifths of State consumption [19]. Of all the states, Texas has the largest crude oil production and proved reserves in the nation with about 4,944 million barrels [20].

**Figure 4.**
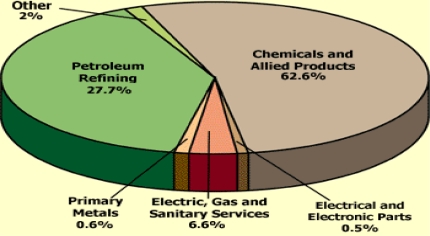
Generation of Hazardous Waste by Industry in Texas, 2001.

Among the major problems, as shown in Table 1, are the ecological issues facing the oil sector from drilling waste management and risk management planning. While the Table highlights the common problems of the industry, it unveils the growing scale at which oil and gas activities contribute to pollution with insidious threats to the ecosystem. Notable environmental problems consist of large volumes of waste materials made up paper, plastics, wood, glass, and metal, generated by offshore oil and gas operations as well as water contamination [22]. Within the Gulf of Mexico, of the total marine debris tabulated for the three neighboring states of Texas, Louisiana and Mississippi, roughly 66 percent occur in Louisiana and Texas as compared to 34 in Mississippi [23]. On the distribution of hazardous waste generation by industry in Texas, in the 2001 fiscal year, note that chemicals and allied products accounted for 62.6%, the other 27.7% came from petroleum refining. This is much larger when compared with the combined total for the other sectors of the economy at that time (Figure 4).

According to the Railroad Commission of Texas, as of August 2008, there were about 14,415 wells classified as non complaint inactive wells that were in violation of the commissions’ plugging rule. Of the 14,415 non complaint wells, 5,092 wells belonged to the operators with an active organizational report on file and with the commission and 9,323 wells belonged to operators with delinquent organizational reports [24]. While the commission defines these 9,323 facilities as orphan wells, the current regulatory frameworks in the state require operators to plug them at their expense upon the cessation of production. Knowing the sanctions awaiting violators of the plugging rules for non compliance, during 2003 to 2008 the state witnessed the plugging of 8,400 non compliance oil and gas wells (Table 2). With the current level of ecological threats from the petroleum sector on the rise, using GIS technology provides opportunities to assess the impacts of these activities and the spatial dispersions in the face of mounting environmental liabilities in various oil and gas districts of the state.

## 2. Materials and Methods

The methodology of the paper stresses a mix scale approach anchored in geospatial information technologies and the integration of primary data provided through government sources and data bases from other organizations. The raw spatial data and other information used in the research were procured through the EPA, environmental scorecard.org, The Texas Railroad Commission, Texas Commission of Environmental Quality, US Department of Energy, The United States National Aeronautical and Space Administration NASA and the United States Geological Survey.

### 2.1. Step 1: Data Acquisition

The first step involves the identification of the variables needed to assess environmental decline at the state level within oil producing counties. The variables consist of such variables as number of gas producing wells, number of oil producing wells, volume of natural gas production, volume of oil produced, petroleum products associated with CO_2_ emission, such as non LPGs, LPGs, and natural gas, amount of gas flared and vented, amount gas lost during extraction, quantity of CFCs emitted by facilities, the volume of benzene emitted by facilities, quantity of toluene emitted by facilities, the amount of waste generated, quantity of pollution by top 20 emitters and inter state movement of natural gas (See tables 1 and 3). This process continued with the design of data matrices and geographic coordinates for the variables covering the various periods from 1999, 1987 and 1999 and beyond. In addition, to the design stage, access to databases and abstracts that are presently available within the Government archives in Texas counties, The United States National Aeronautical and Space Agency (NASA) and host of other organizations helped facilitate the search process.

### 2.2. Step 2: Geo Spatial Data Acquisition and Processing

The design of spatial data needed for the GIS analysis required the identification of the appropriate digital county boundary lines covering the study periods of 1992, 1997 and 2002. This entailed the assemblage of the electronic version of available oil and gas district maps and resource and land cover maps containing oil producing regions of the state of Texas and those responsible for pollution for the years of 1992, 1997 and 2002.

This was made possible by retrieval of spatial data sets of shape files and grid files from the Mississippi Automated Resource Information System (MARIS) in digital form of ARCVIEW GIS and some from the Texas Railroad Commission. With the official boundary lines between several counties in the state apparently stable, it was possible to assign consistent geographic identifier code to the respective area units in order to maintain analytical coherency.

The remaining procedure involves spatial analysis and output (maps-tables-text) covering the study period, using ARCVIEW GIS. Outputs for the region were mapped and compared cross time. This process helped show the extent of temporal-spatial evolution of ecological change induced by oil and gas activities along the oil producing districts of Texas. Accordingly, GIS technique remains indispensable in the development of the appropriate resource management tools for effective policy making in the ecosystem assessment of oil industry impacts.

## 3. Results and Discussion

To assess the extent of environmental damages, a temporal profile of the core oil and gas variables associated with production, distribution, and consumption is necessary. A time series analysis of the indicators in Table 3 show an increase in gas production, number of wells and recurrent fluctuations in oil production and wells between 1987 and 2007. The percentage of change is also presented in the table. From Table 3, note the number of oil and gas producing wells for Texas and the production history from 1987 to 2007. From the information on the second column on the left, gas producing wells grew from 42,167 to 88,311 at a rate of over 100.9%. With the state average figures estimated at about 48,653 gas wells in 1987–1992, in the ensuing period of 1993 to 1998 the average numbers rose to 54,540 wells. Notable evidence of the growing number of production activities during the later years occurred when the available number of natural gas wells rose from 60,486 in 2000 to 68,488 in 2003. In the periods 2004 to 2006, the state’s gas wells further showed some variations.

Conversely, over the 21 year period under analysis, oil producing wells, appear to have plummeted remarkably, at an average of 173,142. With an opening value of 199,354 oil wells in 1987, the numbers slipped to 153,223 in 2007. In the period 1987–1993, the state put an average of 193,951 oil wells into production but only to fall further from 179,955 to 161,097 between 1994 and 2000. In the remaining seven years that span through 2001–2007, Texas’s number of oil producing wells fell drastically. In terms of the total production of natural gas, the state posted some visible increases of 5,516,224,229–6,421,374,997 million cubic feet (mcf) between 1987 and 2007. During the years 1999 through 2000, the state’s production of gas grew from 5,538,929,430 mcf to 5,645,792,009 mcf. In other years, natural gas production went from 5,611,957,703 in 2002 to 5,671,689,242 in 2003. From the table the amount of crude oil production dropped from 725,029 to 336,222 million barrels (mlb) between 1987 and 2007 (Table 3).

The data in Table 4 lists the quantities of petroleum sources of CO_2_ emission for Texas in the categories of petroleum products classified as non liquefied petroleum, non LPs, LPGs and natural gas. From the information as outlined in the table, the intensity of petroleum sources of C_O2_ emission picked up steam from 1986 through 2005 with elevated values in 1988 and 1998, 1986–1987. Note that the study area emitted a combined total of 389,979,421, and 391,000,025 tons of carbon dioxide in 1986–1987. The intensity of petroleum sources of CO_2_ emission soared with sizable value of 415,000,089 to 493,139,066 between 1988 and 1997. Within the period, the volume of pollutants estimated for 1998 exceeded the scale of the previous years. Between 2000 and 2003, the total value of petroleum sources of carbon dioxide jumped further only to stabilize at mid 1990s levels during 2004–2005.

At the same time, gas flaring and the quantities lost during extraction known to impact the ecosystem grew in the period of 1986 through 2000. Of the total of 5,051,195.126 mcf flared, the quantities stayed under moderate values between 1986 and 1990 until the gradual jump of 3,063,760 and 19,689.129 during the fist years of 1991 and 1992. The amount of gas flared rose from the mid 1990s at 42,037,408 in 1994, 46,182.952 in 1995, 45,382.466 in 1996 and 47,921.837 during 1997.

In 1999 and 2000 when the amount of gas flared moved down to 1990s levels, the quantity of vented gas stood at 35,674.983, 32,009.584 respectively. In the ensuing years, the volume of gas lost during extraction stayed stable. With the total of 42,568,145 mcf of gas moved across state lines, the interstate movement of gas remained active in the state.

During the years, the largest volumes of interstate movement occurred during 1994 to 1997 fiscal years. This was followed by moderate levels of oil and gas shipments between 1987 and 1983. Similar levels of shipments remerged within the remaining 3 years of 1998, 1999, 2000 (Table 5). For a brief analysis of the percentages of changes on the variables herein analyzed in this section, please refer to Appendix A.

**Figure 5.**
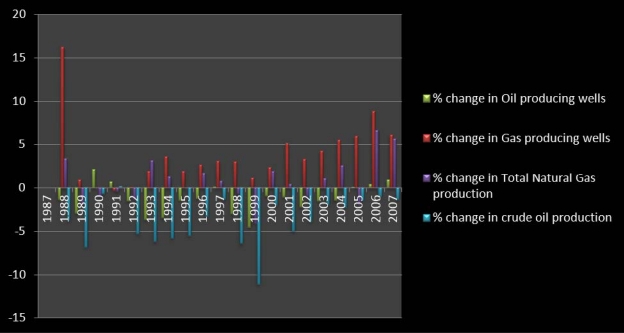
Graphical Summary of the Percentage of Change.

**Figure 6.**
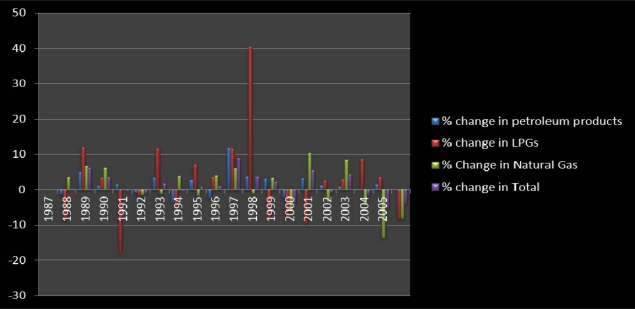
Graphical Summary of the Percentage of Change.

**Figure 7.**
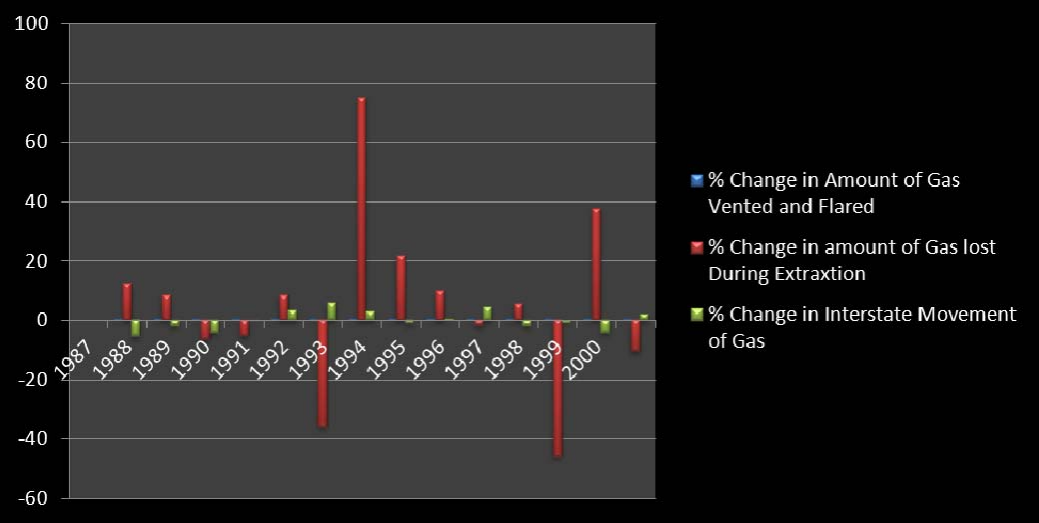
Graphical Summary of the Percentage of Change.

The graphical summary of the analysis presented in this section of the paper are contained in Figures 5–7 on the back of the Tables. The information in Figures 5, 6, and 7 offers a graphical summary of the percentages of change as described in the temporal analysis already presented. The vertical axis of the graph highlights the periods of growth in the percentages of change while the low or horizontal axis distinguished in negative signs point to declines. From the graphical snapshots in Figure 5, 1987–1988 in deep red emerged as a period with the largest percentage increase in the number of gas producing wells as indicated in Table 3. On the other axis of the graph, the 1998–1999 period in blue represents the period of a much higher level of decline estimated at −11% than the other periods. In Figure 6, the highest points in the graph indicating the emission of LPG seemed more visible during 1997–1998 at 40%, followed by 11.03% on the horizontal axis in 1990. The things that stand out in Figure 7 are the colossal percentage increases in the amount of gas lost during extraction around 1994 and 2000 (estimated at 77%, 37% respectively). This was followed by 1993 and 1999 when the amount of gas lost during extraction showed highest levels of decline. For additional explanation describing, the analysis of the trends behind the fluctuations, refer to Appendix B.

In a survey of solid waste generation among several industries in 2002 as presented in Table 6, the oil and as gas sector led the list with 82.9 million tons of waste in terms of ranking. Of the top 20 emitters in Table 7 based on criteria pollutants, the oil sector was ranked 14th, 16th, 17th and 19th based on criteria pollutants (SO_2_ and NO_2_) in 2000. A break down at the county level point to Exxon Mobile Oil in Jefferson County with the largest volumes of pollutants estimated at 29,012 tons.

Other hazardous items from petroleum industry related facilities in the study area consist of ozone depleting substances made up of carbon tetrachloride and large portions of benzene responsible for cancer hazards. The others include the rising emissions of Toluene. From the information on Table 8, facilities releasing ozone depleting chemicals seemed fully spread across the oil producing counties. In terms of the breakdown, the facilities with sizable emissions in order of importance consist of GB Bioscience Corp located in Houston and DDE Beaumont with 55,000 pounds of CFC in 2002. Other notables include BP in Texas City Refinery, and DuPont facilities in Corpus Christi, each responsible for the discharge of 37,000 and 35,000 pounds, respectively. While the Dow chemical facility at Freeport released close to 250,000 pounds, the reaming facilities discharged emissions of meager quantities of CFC compared to the others. The number of petrochemical facilities contributing to the emission of cancer related substances stretches from Beaumont to Baytown. The largest emitters are those in Beaumont, Deer Park, Houston and Freeport. In these areas, about 141,000,000 and 11,000,000 pounds of Benzene were traced to DDE facility in Beaumont and Deer Park Refinery LP.GB Bioscience Houston and Dow Chemical Company in Freeport discharged 8,900,000,000 and 3,100,000 pounds of benzene, equally known to cause cancer in humans. The facilities in the remaining counties ranked 22–35 emitted sizable amounts of benzene lethal enough to harm humans in the area under analysis of all the counties, Baytown and Beaumont had more concentration of such facilities than the others in 2002 (Table 9).

Among the facilities contributing to non-cancer hazards, most notably toluene, the facilities in Houston, Baytown topped the ranking among the petroleum companies. The volume of toluene originating from the facility was quite sizable. Another group of five facilities with sizable discharges of toluene is located at Beaumont, Three Rivers, Freeport, and Sunray counties. The remaining factories include CITGO in Corpus Christi and La Gloria in Tyler County. From the Table, see that the facilities 5 and 6 in Beaumont and Three Rivers each had identical discharge volumes of toluene estimated compared to Freeport and Sunray (Table 10).

### 3.1. Discussion

**Figure 8.**
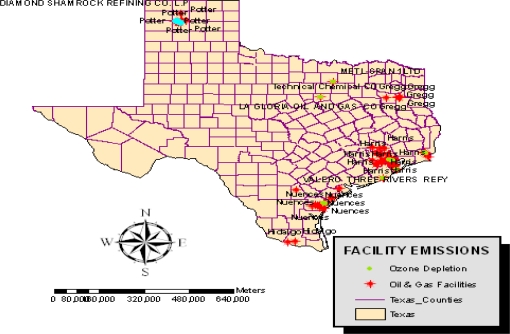
Spatial Distributions of Facilities Producing CFCs.

**Figure 9.**
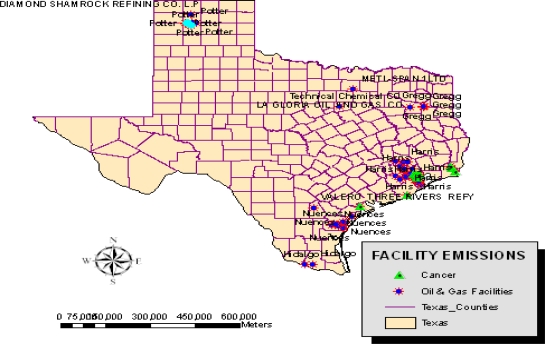
Spatial Concentration of Facilities Emitting Cancer Causing Substances of Benzene

**Figure 10.**
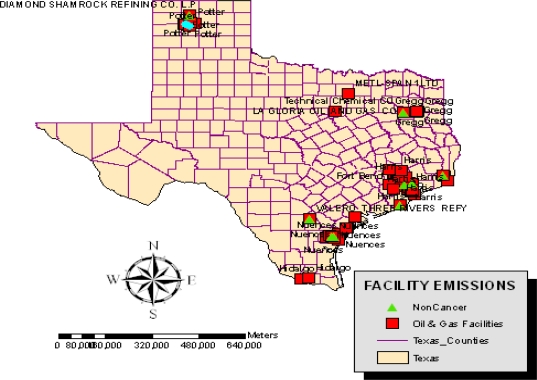
Spatial Concentration of Facilities Emitting Non-Cancer Causing Substances of Toluene.

The preliminary assessment of the extent of change associated with oil and gas activities, which culminated over years, has revealed growing environmental impacts. The gravity of ecological damages in the study area is closely associated with the intense nature of oil and gas activities and a host of other variables. Pertaining to the geographic concentration of facilities responsible for the emission of ozone depleting substances, CFC and those discharging cancer causing chemicals containing benzene, the maps in Figures 8 and 9 show a patchy presence of those companies along the southeastern part of the state distinguished in blue and red colors. Note also the presence of companies responsible for the emission of non-cancer substances most notably large quantities of toluene along the southern tip of Texas (Figure 10).

**Figure 11.**
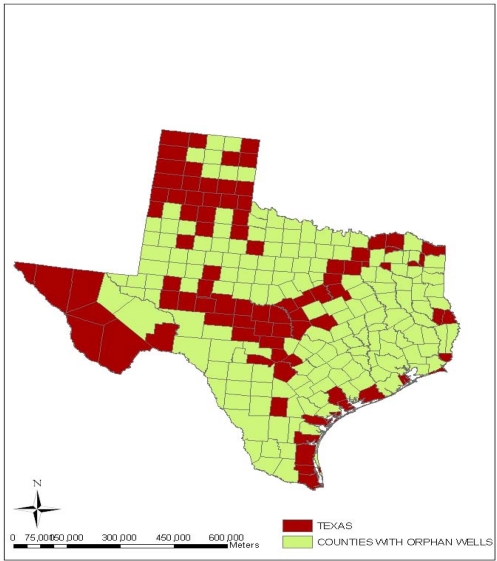
Spatial Distributions of Orphan Wells in Texas Counties.

**Figure 12.**
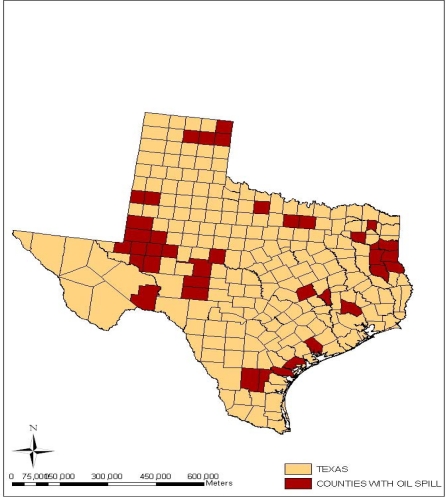
Counties in Texas with Oil Spill Occurrences.

Additionally, Figure 11 provides an indication of the geographic distribution of orphaned wells in the state of Texas. In the map notice a strong cluster of wells along the upper north and western portion of the state. From the map, it is evident that there are very large collections of orphaned wells in the central and coastal areas of the state. Because abandoned oil wells of this proportion serve as conduits for the seepage of brine, salt water and other toxic fluids, the upper portions of the Colorado River adjacent to the state’s area have been severely degraded in last several years. As part of the consequences, rural residents were compelled to evacuate areas deemed adjacent to abandoned wells and saltwater disposal pits [22]. Another interesting twist to the type of impacts being experienced in the state of Texas is the growing incidence of oil spills and oil well blow outs. While oil spills as presented in Figure 12 appear somewhat sketchy when compared to abandoned wells note that oil spill incidents seemed scattered in various counties of the state. There appears to be a sizable concentration on the areas located around the North East, the South East corner of the state as well as the south west. From the map there exists a close proximity between some of the counties experiencing oil spills and the surrounding costal environment of the state.

**Figure 13.**
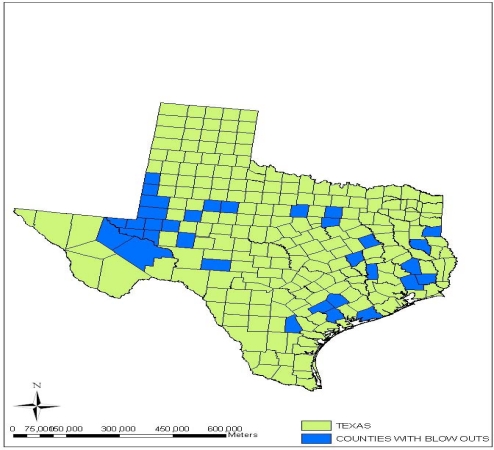
Blowout Occurrences in the Counties of Texas.

**Figure 14.**
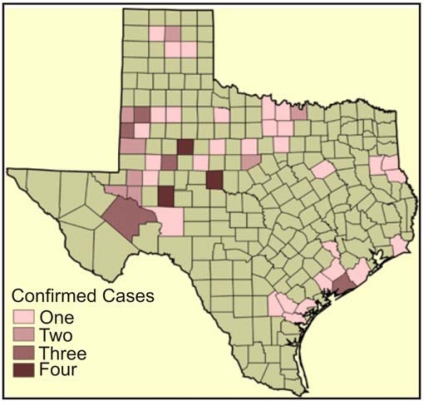
Texas Counties with Cases of Underground Water Pollution Due to Oil and Gas Activities.

Regarding the number of blow out incidents from oil and gas wells in the state of Texas, the information as presented in Figure 13 indicates a sizable dispersion of the problem in various counties. The northwest section of the state has strong concentration in space, while much of it is scattered in the south-eastern part and the coastal areas. In terms of sub-terrain pollution, Figure 14 offers a vivid example of some of the counties in the state of Texas experiencing confirmed cases of underground water contamination due to oil and gas activities between 1997 and 1998. The spatial distribution of ground water contamination from the map shows that it transcends every geographic location of the state. According to the map, the first three confirmed case types of ground water contamination were quite pronounced along the coastal and Southern plain of the state of Texas known for high concentration of petroleum refineries. The north central portion of the map as shown in the Figure 14 contains a cluster of confirmed cases of 1 and 2 along with the maximum case types of 4 as well. All in all, the North Central and North West part of the state have far more cases of underground pollution when compared with the southern portion along the Gulf coast of Texas. This problem poses enormous health risks for humans and animals that depend upon underground water supply in the area. For more discussions on the spatial analysis see Appendix B.

The ecological impacts of oil and gas activities in Texas do not operate in a vacuum; they are associated with several variables. The factors center on demography, economic indicators and policy defects. This section of the paper analyzes the factors fueling the growing impacts of oil and gas activities and inaction towards periodic monitoring of the trends using geospatial information technologies.

#### 3.1.1. Socio-demographics

Over the last several years, the state of Texas has experienced major growth in the number of new residents that require the design of new supply lines and new pipelines to meet the needs of industrial and residential consumers. Meeting the energy needs of a growing population in this setting exerts enormous pressure on the environment. With a population of 24.3 million in 2008, Texas ranks as the second largest state and the number one consumer of oil and natural gas in the country. The design of new cities and the rapid pace of industrial development in the state spurred by intense population growth continue to trigger large demand for oil and gas. The break down of Texas population showed that it grew from 10, 599,000 in 1967 to 13,193,050 in 1977. In the following decade of 1987, the state’s number of residents jumped to 16,621,790. As of 1997, the state witnessed further increases of 19,740,317 coupled with its highest level of 20,851,820 million it attained in 2000. The ensuing influence of population pressure can be seen with vegetation clearance, widespread emissions, flaring and venting of natural gas and other by-product of fossil fuel extraction hazardous enough to fuel ecological degradation [9].

#### 3.1.2. Economic elements

For years the petroleum industry has remained a key component of the economy of Texas. With present policy focused solely on production, oil sector operations involving drilling, geologic and seismic surveys have sizable impacts on biodiversity. Accordingly, there exist several economic indicators in the petroleum sector that are partially responsible for the problems. Under a system in which environmental damages are often omitted from the conventional economic system and core fiscal indicators, in 2006 alone there were about 6,900 active operators, 229,050 producing wells of which oil accounted for 149,100 and natural gas 79,950. The state is ranked the number one producer of oil and natural gas with 348 million barrels of oil and 5.9 TCF of natural gas. With 26% of the United States refinery capacity and 8981 rotary rigs in operation, Texas accounts for 47.8% of US total. The state’s oil and gas operations represent a $100 billion industry. The drilling permits issued in Texas have also risen over the years as indicated in Table 11. In fact, the number went from 9,716, to 12,664 between 2002 and 2003 and rose from 14,700 to 16,914 during the fiscal years 2004 and 2005. In 2006, the demand for permits reached the 21,000 mark [21]. At the same time, the state’s oil and gas Gross Product has been growing in the face rising of environmental liabilities of the sector.

#### 3.1.3. Policy defects

The current lapses in policy seem somewhat associated with the growing levels of oil and gas impacts on the environment and the meager geospatial tracking of the trends in the study area. Under a regulatory climate festooned with major weaknesses and lapses and a large concentration of 244,460,108 pounds of some of the most toxic pollutants, Texas ranks 4th in the nation in regards to environmental decline. Various indicators for gauging environmental decline such as the total environmental releases, air releases of recognized carcinogenic substances, atmospheric discharge of developmental and reproductive toxicants, air and water releases remain so threatening that they have been classified as the worst among the 50 states. According to the state summary of emissions, in 2000, 100 facilities in Texas emitted into the atmosphere more than 38,000 tons of particulate matter less than 10 microns along with 794,000 tons of sulfur dioxide, 497,000 tons of nitrogen oxides, 76,000 tons of non-methane organic compounds—including all VOCs—and 224,000 tons of carbon monoxide [25]. Because many of these emission sources or facilities were built before 1972, they escaped major air pollution control requirements under the 1972 Texas Clean Air Act until recent changes were enacted by the 1997, 1999 and 2001 Legislatures. These facilities are known as grand fathered facilities. The weaknesses in regulatory framework is compounded further by the loosely defined land use policies and the limited emphasis on periodic assessment of the impacts of oil and gas activities using the latest advances in geospatial information technologies.

Realizing the scale of oil and gas activities and the impacts on the environment, numerous initiatives have been undertaken by various entities in the state. This part of the project describes the efforts put into place. Some of the efforts consist of legislation to plug abandoned wells, to invoke operator clean up programs, to propose acquisition of new technologies and to promote stakeholder initiatives.

#### 3.1.4. Plugging legislations

The oil field clean up came into effect after its approval through the Senate Bill (SB 1103) and revision contained in SB 310 by the legislature in 2001. In line with the provisions of SB 1103, the State of Texas along with the Railroad Commission, reinforced its fiscal capacity to close abandoned, orphaned oil and gas wells through immediate rehabilitation and restoration of affected oil field sites. In the process, SB 1103 replaced the previous well plugging fund with the oil field clean up account by setting the fund balance cap at $10 million and above. The effect of the oil and field clean up fund remains evident given the growing number of plugged orphaned wells and rehabilitated sites. In the beginning of the financial year 1984 to 1991, the commission plugged 4,078 wells with an estimated price tag of $16.1 million made possible through an earlier well plugging fund. Additional work involving the commission include the plugging of 24,797wells at a cost of $139,574,743 during the financial the year 1992 through fiscal year 2008 as well as numerous efforts directed at cleaned up, assessment and monitoring of 3,983 sites with money from the state, federal sources and the oil clean up of fund [24].

#### 3.1.5. Industry role in Operator Clean Program (OCP)

Ever since the financial year 1992, the Railroad Commission and the industry have partnered to plug about 6,000 and 10,000 wells annually. Consequently, the numbers of orphan and non compliant wells have declined in the last four years. One more vital role of the commission’s Oil Field Clean up Program revolves around the administration of the operator Clean Up Program. Operator clean ups involves multifaceted appraisal and rehabilitation on initiatives carried out by a dependable operator, typically at ecologically fragile sites. The plan stipulates that pollution intense operations far-off SWR 91 non-fragile area adhere to oil spill clean up rules and beyond SWR 8 and that regular clean ups and closings be dealt with on time and cleaned up satisfactorily. Supervision of OCP activities is generally done through employees in Austin headquarters and District office (DO) staff where the bulk of long-standing remediation plan call for expert skills to appraise and administer. For more information on the extent of clean up activities in selected counties see Table 12 [24].

#### 3.1.6. Information technology acquisition

The project centers on sustaining the Railroad Commission efforts in designing a manageable and secured computing environment. The project will tackle end use computing software, printer replacement, mobile computing, security and network improvement required to sustain current technology infrastructure. The project will change obsolete network hardware and procure software that will enhance the rail road commission capacity to manage network activity and strengthen infringement recognition ability. While some components of the Railroad Commission computing infrastructure have been replaced during the biennium, other infrastructure improvements will be needed during FY10-FY11 biennium. These improvements are pertinent for the end user computing software and peripheral replacement and network improvement. Under the program, secondary tools including scanners, cameras, GPS devices, external storage unit and drives to combat device failure are of topmost priority [26].

#### 3.1.7. Support for innovative approaches

Both government and industry have long recognized the importance of cost efficient approach to environmental safety in the oil sector. This realization prompted the establishment of ONGPTs oil and gas environmental research and analysis program. The national petroleum council at the insistence of the secretary of energy highlighted various ways upon which government and industry might partner jointly to fulfill this requirement. Among the council’s suggestions were the formulation of a less rigid policy and regulatory structure along with more proficient remediation tools to lessen environmental effects and good science. The expectation is that this would give oil and gas producers more flexibility in determining how they can best meet standards, yielding the same environmental benefits at lower costs [8].

#### 3.1.8. Multiple stakeholder efforts

Numerous stakeholders are playing active roles to address the growing environmental problems from oil and gas operations. The Department of Energy is working closely with industry, states officials, and the other federal agencies to stem the rising costs of environmental protection. This is intended to help oil and gas producers operate more effectively and generate jobs and economic activities of value to the nation. DOE together with state officials and leaders from the oil and gas industry are using the best information and science available to find new ways to address the nation’s environmental concerns. The results of their collaboration demonstrate that the needs of a strong economy and healthy environment can indeed be fully compatible. While some of the environmental NGOs and community groups and centers of learning in the state have been quite active in raising the profile of environmental impacts of oil and gas activities, others have focused their efforts in the areas of research and development to stem the tide of ecological decline emanating from the oil and gas sector.

Aside from the efforts of policy makers and stakeholders through policy and legislations to mitigate the problems of pollution emanating from oil and gas activities, the state faces a daunting task in eradicating the threats posed by petroleum producing facilities located in several counties of the state of Texas. With the large presence of indicators of environmental decline in most production facilities, the surrounding ecology of the oil producing districts remain under serious stress. Additionally, a time series analysis of the trends indicate an increase in gas production and number of wells followed by recurrent fluctuations in oil producing wells and the quantity produced between 1987 and 2007.

In the context of the ecosystem health of the state, the aforementioned problems can prolong the current threats of environmental degradation through growing air pollution, ozone depletion, rise in climate change factors, water quality decline, high cancer rates and other health related complications among the communities along with coastal ecosystem change and the large presence of hazardous waste materials.

To deal with some of the problems herein identified, the paper suggests four major future lines of action. The measures include the need to encourage the continuous use of geospatial information technologies, collaboration with the industry, improvement in current policy and the need for the development of energy information system and the involvement of communities.

#### 3.1.9. Encourage the regular use of spatial information

With the result of the research showing a widespread cluster of pollution intense facilities in selected counties of the state and the proximity of such facilities to sensitive natural systems. The assessment of ecosystem health of counties adjacent to the operation of petroleum facilities and the spatial dispersion of the trends are better done using GIS technology. As a valuable decision support tool for mitigating the threats posed by oil and gas externalities, geo-spatial information technologies enables researchers and decision makers to detect the dangers posed by impending changes. They not only enhance our understanding of their scale, but they offer a framework for evaluating ecosystem decline and the mechanisms for restoration. The responsible use of natural resources in an ecosystem especially along sensitive or costal areas involves also effective geospatial monitoring with GIS. With environmental surveys costly especially in remote areas of large tracts including water bodies and oil and gas wells. Integrated remote sensing and GIS have significant potential to aid environmental monitoring/change detection efforts.

#### 3.1.10. Improve current policy

The scale of problems occurring in the oil producing areas of Texas, calls for an urgency to improve the current policy. This can be attained by strengthening the regulatory instruments with stringent requirements on standards, licensing along with mandatory disclosures and reporting of activities. Such measures by the governments would not only help address the discharge of pollutants and environmental degradation, but will go a long way to streamline policy goals focused on the welfare of the ecosystem based pollution reduction.

#### 3.1.11. Develop regional energy information system

During the writing of this research paper, much of the information needed for the project were scattered in various agencies including state, federal, academic institutions, and environmental NGOS. In order words, we researched the impacts of petroleum activities on a major oil producing state with access to no centralized regional energy information system with geospatial orientation. The design of such a system offer a creative approach in dealing with environmental problems emanating from the industry by providing up to date access on information highlighting the state of the resource, the impacts and indicators in order to ensure the sustainable management of fossil fuel products in the oil producing districts of Texas. To improve decision making capability and provide support, the proposed system would offer periodic display of information on the location of delinquent wells, compliance patterns among operators, the number of wells in operation and the interaction between the activities of the sector and the surrounding ecology and the ability of the regulators able to predict and monitor the impacts.

#### 3.1.12. Involve communities in the management of the natural resources

The problems associated with the impacts of oil and gas activities are so localized and some times regional with much of effects often felt in the neighboring communities. At the same time, unhealthy environments make residents more vulnerable to mortality from diseases. As a result, many communities in whose domain the petroleum facilities operate remain at the center of activities and on the receiving end in terms of exposure to environmental burdens, high cancer rates and water contamination. The belief is that local populations have a greater interest in the sustainable use of resources than others and that local people are more cognizant of the intricacies of local ecological process and practices. Using community-based strategies to optimize environmental initiatives in the oil industry have potentials to improve the quality of life and the ecosystem health.

## 4. Conclusions

This paper has presented the use of spatial technologies in environmental management by analyzing the case of ecosystem assessment of oil and gas activities in Texas. The paper outlined an overview of the background with some focus on the ecological issues in the literature pertaining to oil and gas production in the state, the essence of Geospatial Information System and the benefits of the petroleum sector to the economy. This was followed with the outline of the profile of the study area with some emphasis on the pollution threats within the oil and gas districts and the description of the GIS techniques and methods used, the analysis of environmental impacts of the petroleum sector, the factors fueling the problems and mitigation efforts. The results point to widespread growth in indicators of environmental decline attributed to oil and gas activities in the area.

With the growing presence of pollution indicators from the oil sector, mix-scale approach involving descriptive statistics and GIS applications point to a rise in number of gas producing wells and production with recurrent drops in number of oil wells and quantity produced. While there were increases in petroleum sources of CO_2_ emissions coupled with gas flaring, the amount of hazardous waste materials and other emissions attributed to the sector remained sizable. Other hazardous chemicals consistent with the petroleum producing facilities in the study area such as ozone depleting substances made up of carbon tetrachloride and large portions of benzene responsible for cancer hazards as wells as the rising emissions of toluene pose serious threat to the ecosystem health of the communities.

The geospatial assessment of the impacts using GIS, reveal visible cluster of facilities responsible for the emission of ozone depleting substances, CFC and those discharging cancer causing chemicals containing benzene and non cancer toluene materials along the South East part of the state. Geospatial analysis of impacts, offered a valuable insight on the location and risks associated with vast number of orphaned wells in the central and coastal areas and the risks. The presence of numerous abandoned oil wells as the analysis showed create easy conduits for the seepage of toxic materials into ground water. These substances appeared more pronounced on areas situated around the northeastern, southeastern, as well as the southwestern corner of the state. The GIS mapping which highlighted the spatial distribution of oil spills indicates a close proximity between oil spills sites and fragile costal environment of the state as well. There were numerous cases of blow out incidents from oil and gas wells and ground water contamination in various counties of the state of Texas as well.

In the current study, the availability of temporal spatial data and analysis played a vital role in facilitating the assessments of the ecological risks emanating from the oil sector. The assemblage of the information and analysis as an emerging science devoted to the study area, not only quickened the data processing stage of the study, but it unveiled the location of stress indicators in the state essential to effective management and timely mitigation. Accordingly, GIS technique, as used here in contributing to the literature, stands as a relevant decision support tool that pin points high risk areas threatened by oil and gas externalities. In the study area, this involved the generation of maps that identified externalities such as oil and chemical spill, well blow out incidents, ground water contamination along coastal areas and the spatial distribution of facilities producing CFCs, cancer causing substances of benzene and toluene and orphan wells. Visualizing natural systems prone to disasters from the oil and gas sector in these settings, not only helped focus the scope of environmental management with records of change in affected areas, but it furnished information on the pace at which resource extraction activities impact nature.

For the purposes of planning, spatial analysis offered a visual documentation of environmental health at precise locations on different sets of variables related to oil and gas activities in Texas. With the capability to generate temporal spatial information, this perspective serves the needs of managers in weighing the significance of the emerging patterns and the impacts on the local ecosystem. Without access to such information, resource and environmental managers run the risk of offering improper blue prints and solutions for protecting the environment. GIS applications can be effective as part of an emerging science for addressing these concerns by providing managers a yardstick for analyzing different levels of changes in the ecosystem. It is expected that they will serve a useful purpose in subsequent research and will evolve further through utilization in a variety of situational settings in the study area and elsewhere under conditions that are compatible with the ideas of environmental management.

The study also serves as a conduit for future applications in oil producing areas in states or regions. This then stimulates the growth of regional expertise and confidence which in turn enhances the capacity to make decisions in areas associated with petroleum exploration and ecosystem impacts. This role as a decision support tool, can lead to a real consensus as more users, and those in charge of environmental management systems have faith in the approaches and make a conscious decision to increase their application in future. The applications of this technique in the research along with the findings from it therefore make a contribution to our understanding of GIS applications in environmental management. These techniques play a fundamental role with the steps upon which impact analysis of petroleum activities is built. The project has revealed the utility of GIS applications in environmental management and thus serves as conduit for future applications as novel science in communities impacted by oil and gas activities.

## Figures and Tables

**Table 1.** Common Environmental Problems of the Oil Industry.

**Industry Issues**	
Drilling waste management	Spill prevention
Low impact operations in sensitive environments	Remediation
Public lands/leasing	Air emissions, Toxic releases
Produced water management	Underground injection
Production waste management	Risk management planning

Note: DOE 1997.

**Table 2.** Number of Non Compliance Wells Plugged.

**Years**	**Numbers**
**2003**	**1,527**
**2004**	**1,726**
**2005**	**1,756**
**2006**	**1,877**
**2007**	**1,514**
**2008**	**1,143**

**Total**	**8,400**

Note: RRCT 2008 [24].

**Table 3.** Number of Oil and Gas Producing Wells for Texas 1987–2007 and Percentages of Change.

**Year**	**# Gas Producing Wells**	**# Oil Producing Wells**	**Total Natural Gas Production MCF**	**Crude Oil Production (Mbbl)**	**% change in Gas producing wells**	**% change in Oil producing wells**	**% change in Total Natural Gas production**	**% change in crude oil production**
**1987**	42,674	199,354	5,516,224,229	725,029				
**1988**	49,577	196,580	5,702,643,736	698,224	16.17	−1.39	3.37	−3.69
**1989**	50,017	190,821	5,595,189,980	650,514	0.88	−2.92	−1.88	−6.83
**1990**	49,989	194,962	5,533,770,539	645,941	−0.05	2.17	−1.09	−0.70
**1991**	49,825	196,292	5,509,989,547	646,776	−0.32	0.68	−0.42	0.12
**1992**	49,839	193,310	5,436,407,608	612,692	0.02	−1.51	−1.33	−5.26
**1993**	50,794	186,342	5,606,497,721	574,568	1.91	−3.60	3.12	−6.22
**1994**	52,614	179,955	5,675,748,270	541,482	3.58	−3.42	1.23	−5.75
**1995**	53,612	177,397	5,672,105,129	511,962	1.89	−1.42	−0.06	−5.45
**1996**	55,052	175,277	5,770,254,648	495,378	2.68	−1.19	1.73	−3.23
**1997**	56,736	175,475	5,814,744,733	488,860	3.05	0.11	0.77	−1.31
**1998**	58,436	170,288	5,772,079,829	457,499	2.99	−2.95	−0.73	−6.41
**1999**	59,088	162,620	5,538,929,430	406,815	1.11	−4.50	−4.03	−11.0
**2000**	60,486	161,097	5,645,792,009	398,678	2.36	−0.93	1.92	−2.00
**2001**	63,598	159,357	5,668,602,291	378,849	5.14	−1.08	0.40	−4.97
**2002**	65,686	155,865	5,611,957,703	364,314	3.28	−2.19	−0.99	−3.83
**2003**	68,488	153,461	5,671,689,242	357,240	4.26	−1.54	1.06	−1.94
**2004**	72,237	151,205	5,817,226,749	349,233	5.47	−1.47	2.56	−2.24
**2005**	76,510	151,286	5,700,612,714	344,226	5.91	0.05	−2.00	−1.43
**2006**	83,218	151,832	6,077,785,935	340,885	8.76	0.36	6.61	−0.97
**2007**	88,311	153,223	6,421,374,997	336,222	6.12	0.91	5.65	−1.36

**Table 4.** Temporal Distribution of Petroleum Sources of Carbon Dioxide Emissions and Percentage of Change.

	**Quantities of Petroleum Sources of CO**_**2**_**Emission For Texas**				**Percentages of Change**			
	
**Years**	**Petroleum Products (Non−LPG)**	**LPGs**	**Natural Gas**	**Total**	**% change in petroleum products**	**% change in LPGs**	**% Change in Natural Gas**	**% change in Total**
**1986**	192,938,722	25,768,442	171,272,257	389,979,421				
**1987**	190,289,171	23,487,865	177,222,989	391,000,025	−1.37	−8.85	3.47	0.26
**1988**	199,637,991	26,296,765	189,073,333	415,008,089	4.91	11.95	6.68	6.14
**1989**	201,823,490	27,181,450	200,476,367	429,481,307	1.09	3.36	6.03	3.48
**1990**	204,815,884	22,388,967	200,700,067	427,904,918	1.48	−17.63	0.11	−0.36
**1991**	203,269,872	21,974,549	197,852,336	423,096,757	−0.75	−1.85	−1.41	−1.12
**1992**	210,051,432	24,517,669	195,452,383	430,021,484	3.33	11.57	−1.21	1.63
**1993**	202,561,891	23,168,625	203,014,985	428,745,501	−3.56	−5.50	3.86	−0.29
**1994**	207,823,879	24,786,272	199,645,019	432,255,170	2.59	6.98	−1.65	0.81
**1995**	203,290,526	25,639,078	207,697,666	436,627,270	−2.18	3.44	4.03	1.01
**1996**	227,178,365	28,602,183	219,927,161	475,707,709	11.7	11.55	5.88	8.95
**1997**	235,524,964	40,128,100	217,486,002	493,139,066	3.67	40.29	−1.10	3.66
**1998**	242,387,811	36,919,685	224,530,757	503,838,253	2.91	−7.99	3.23	2.16
**1999**	236,979,534	33,915,563	211,599,786	482,494,883	−2.23	−8.13	−5.75	−4.23
**2000**	244,398,369	30,679,121	233,416,399	508,493,889	3.13	−9.54	10.31	5.38
**2001**	247,214,901	31,423,138	225,434,330	504,072,369	1.15	2.42	−3.41	−0.86
**2002**	249,386,408	32,302,526	244,275,064	525,963,998	0.87	2.79	8.35	4.34
**2003**	248,927,290	35,088,462	233,631,225	517,646,977	−0.18	8.62	−4.35	−1.58
**2004**	252,577,214	36,280,757	202,006,712	490,864,683	1.46	3.39	−13.53	−5.17
**2005**	252,703,344	33,265,551	185,611,785	471,580,680	0.04	−8.31	−8.11	−3.92

**Table 5.** Environmental Change Indicators during Oil and Gas Activities in Texas and Percentage of Change.

**Year**	**Amount of Gas Vented and Flared**	**Amount of Gas Lost During Extraction**	**Interstate Movement of Gas**	**% Change in Amount of Gas Vented and Flared**	**% Change in amount of Gas lost During Extraction**	**% Change in Interstate Movement of Gas**
**1986**	26050	384693	3093141			
**1987**	29325	364477	2929521	0.05	12.57	−5.25
**1988**	31832	357756	2949238	0.05	8.54	−1.84
**1989**	29770	343233	2922632	0.05	−6.47	−4.05
**1990**	28247	342186	2883075	0.05	−5.18	−0.30
**1991**	30637.698	353737	2742234	0.05	8.46	3.37
**1992**	19689.129	374126	2748086	0.05	−35.73	5.76
**1993**	34486.27	385063	2571929	0.05	75.15	2.92
**1994**	42037.408	381020	2672096	0.05	21.89	−1.04
**1995**	46182.952	381712	3203828	0.05	9.86	0.18
**1996**	45382.466	398442	3413156	0.05	−1.73	4.38
**1997**	47921.837	391174	3172315	0.05	5.59	−1.82
**1998**	25948.799	388011	2562270	0.05	−45.85	−0.80
**1999**	35674.983	372566	2374107	0.05	37.48	−3.98
**2000**	32009.584	380535	2330517	0.05	−10.27	2.13

**Table 6.** Generators of Hazardous Waste in Texas.

**GENERATOR**	**Tons**
Industry: Hazardous Waste	63 Million
Industry, Oil and Gas Exploration and Development: Non-Hazardous Waste (Class 1 Waste)	82.9 Million
Institutional, Residential, and Commercial: Municipal Waste Recycling	15.7 million

**Table 7.** Emissions Inventory: Top 20 Emitters of Criteria Pollutants in 2000 (In Tons).

**Rank**	**Facility**	**County**	**SO**_**2**_	**NOX**	**TOTAL**
**14**	ExxonMobil Oil Corporation – Beaumont Refinery	Jefferson	16,304	7,342	29,012
**15**	TXU Electric Co. – Sandow Steam Electric	Milam	19,562	6,801	27,561
**16**	Shell Oil – Deer Park Plant	Harris	9,322	7,194	23,689
**17**	British Petroleum – Amoco –Texas City Refinery	Galveston	6,871	7,213	22,225
**18**	Central Power and Light – Coleto Creek Power Station	Goliad	14,721	5,804	21,467
**19**	Phillips 66 Co. – Borger Refinery	Hutchinson	8,550	3,147	20,559
**20**	San Miguel Electric Co. – SM Electric Plant	Atascosa	12,226	7,107	19,837

**Table 8.** Facilities Releasing Ozone Depleting Chemicals: Top Ranked Chemical Carbon Tetrachloride.

**Ranking**	**Facility**	**Location**	**Pounds of CFC 11 Equivalents**
**1**	Metl-span 1ltd	Lewisville	**65,000**
**2**	GB Biosciences Corp	Houston	**55,000**
**3**	DDE Beaumont Plant	Beaumont	**55,000**
**4**	BP Texas City Refy	Texas City	**37,000**
**5**	DOW Chemical Co Freeport Facility	Freeport	**25,000**
**6**	DU Pont Corpus Christi Plant	Gregory	**35,000**
**7**	DOW Chemical Co, Laporte	LA Porte	**2,600**
**8**	Technical Chemical Co	Cleburne	**330**
**9**	Global Octane Corp	Deer Park	**260**
**10**	Occidental Chemical Corp	Gregory	**240**
**11**	Exxon Mobil oil Beaumont	Beaumont	**210**

**Table 9.** Facilities Contributing to Cancer Hazards.

**General Rankings Among Other Companies**	**Facility**	**City**	**Pounds of Benzene Equivalents**
**2**	DDE Beaumont Plant	Beaumont	**14,000,000**
**11**	Dow Chemical co Freeport Facility	Freeport	**3,100,000**
**22**	Union Carbide Corp. Seardrift Plant	Seadrift	**410,000**
**24**	Exxon Mobil Refining and Supply Baytown Refy	Baytown	**310,000**
**28**	Basf Fina Petrochemicals L.P	Port Arthur	**200,000**
**30**	Equistar Chemicals Bayport Chemical Plant	Pasadena	**190,000**
**32**	Millennium Chemicals Inc La Porte Plant	La Porte	**180,000**
**34**	Exxon Mobil Baytown Olefins Plant	Baytown	**170,000**
**35**	Exxon Mobil Baytown Chemical Plant	Baytown	**160,000**

**Table 10.** Facilities Contributing to Non-cancer Hazards.

**Rankings Among Petrochemical Companies**	**Facility**	**Location**	**Pounds of Toluene Quantity equivalents**
**1**	Lyondell-Citgo Refining L.P	Houston	1,900,000,000
**2**	Exxon Mobil Oil Beaumont Refy	Baytown	1,200,000,000
**4**	Exxon Chemical Co Baytown Olefins Plants	Baytown	1,100,000,000
**5**	Exxon Mobil Oil Beaumont Refy	Beaumont	580,000,000
**6**	Valero Three Rivers Refy	Three Rivers	530,000,000
**7**	Dow Chemical Co Freeport Facility	Freeport	420,000,000
**8**	Diamond Shamrock Refining Co. L.P	Sunray	310,000,000
**9**	Citgo Refining and Chemicals Co LP West plant	Corpus Christi	270,000,000
**10**	La Gloria Oil and Gas Co	Tyler	220,000,000
**11**	Citgo Refining and Chemicals Co LP East Plant	Corpus Christi	170,000,000

**Table 11.** The Distribution of Oil and Gas Permits 2002–2006.

**Years**	**Number of Permits**
2002	9,716
2003	12,664
2004	14,00
2005	16,914
2006	21,000

Note: RRCT (2006) [24].

**Table 12.** Voluntary Clean Up Program in Selected Counties from 2004–2008.

**Counties**	**Size of Acreages**
Aransas	90.394
Gregg	108.9
Harris	1291.392
Montgomery	3160.42
Nuences	192.651
Hidalgo	5.043
Galveston	302
Fort Bend	845.27
Potter	1.67

RRCT (2008) [21].

## Appendices

### Appendix A. The Percentages of Change in Oil and Natural Gas

In terms of the percentages of change in gas producing wells as shown in Table 3, with the exception of two periods from 1987–2007, the number of gas producing wells were very much on the rise most of the time. Note that between the opening years of 1987–1988, oil producing wells grew to all time high of 16.17% and in the ensuing periods of 1988–1989, the growth rate stood at 0.88%. This was followed by miniscule drops of −0.05% to −0.32% from 1989–1990, 1990–1991 and by 1991 and 1992 the number of oil producing wells rose by less than 1% or 0.02%. The growth in the available number of gas producing wells all through 1992 to 1999 as the Table shows stayed under 3 percentages points. On the individual periods of 1992–1993 through 1994–1995 and 1996 under analysis, natural gas wells under operation grew by .1.91% to 3.58% during 1992 to 1993 and 1993 to 1994. As the time went by (during 1994 to 1995, 1995 to 1996), the rate of gas producing wells under operation jumped from 1.89% to 2.68%.

Towards the closing decade as the Table shows, the growth patterns stayed at low single digits of 3.02% to 2.99% and 1.11% to 2.36% in the ensuing years of 1996–1997, 1997 –1998, 1998 –1999 and 1999–2000. In the remaining 7 year period of 2000 –2007, when the gas producing wells in the state of Texas grew more than in other periods. The rate of change soared further from 5.14% to 3.28% and 4.26% respectively between 2000–2001, 2001–2002 and 2002 through 2003. In the closing years, during the first the decade of the 21st century, gas production rate moved up to 5.47%–5.91% in 2003–2004 and 2004–2005 and in 2005 through 2007, the number of gas producing wells grew further at a rate of 8.76% and 6.12% respectively. Note also that the percentage of change in oil producing wells in the study area has been on gradual decline during the years. From the Table, the rate of change on oil producing wells plummeted to −1.39% and −2.92% between 1987–1988 through 1988 –1989 until it picked up slightly at a rate of 2.17% and 0.68% during 1989–1990 and 1990–1991.

Beginning in 1992–1996, the study area experienced back to back declines of −1.51%, −3.60%, −3.42%, −1.42%, −1.19% respectively. In the later years of 1997–2007 when the number of oil producing wells, appeared somewhat weak or less than 1 percentage points, the study area experienced recurrent rates of decline. The most visible rates of decline were the −2.95%, 4.50% and 2.19% that surfaced in the area during 1997–1998, 1998–1999 and 2001–2002 fiscal years. In comparing, the percentages of change in total natural gas and crude oil production in each of the 20 year period. It is evident that total natural gas production grew most of the time in 13 of the 20 years under analysis with occasional drops in 7 different periods. Crude oil production in the area on the other hand, dropped regularly in 19 of the 20 years. In 1987–1998, the total gas production grew by 3.37% only to drop to −1.88%, −1.09%, −0.42% and −1.33% respectively between 1988–1989, 1989–1990, 1990– 1991 and 1991–1992. Note also that the growth levels of 3.12% to 1.23% evident in Texas between 1992–1993 and 1993–1994 were followed by intermittent soft declines of −0.06% to −0.73% during 1994–1995 and 1997–1998, coupled with a visible drop of −4.03% during 1988–1999. The seemingly partial declines that occurred in total gas production rates were offset by the recurrent increases of 1.06%–2.56%, 6.61% and 5.65% that emerged in 2002–2003, 2003–2004, 2005–2006 and 2006–2007.

Just as mentioned before, the percentage of crude oil production has been on constant decline. The display of figures on the table show the initial skids estimated at −3.68%, −6.83% and −0.70% during the periods of 1987–1988, 1988–1989 and 1989–1990. All these happened despite the soft increase of 0.12% posted by the study between 1990–1991. From then on as the Table shows, the recurrent drops in crude oil production during 1992–1997, were in the form of −5.26%, −6.22%, −5.75%, −5.45%, −3.23%, −1.31% respectively. The other notable evidence of declines in crude oil production came be seen with −6.41%, 11.0%, −2.00%, −4.97%, −3.83% the study area experienced in the latter years of 1997–1998, 1998–1999, 1999–2000,2000–2001 and 2001–2002. The temporal patterns of change or decline in the closing years of 2002–2003 through 2004–2005 and 2006–2007 were not only stable, but to some degree they reflect the trends over the years in the area (Table 3).

The individual percentages of change for quantities of petroleum sources of emission rose moderately between 1986 through 2005 as the table showed. Accordingly, petroleum products grew 13 of the 20 years while the other variables from LPGs to percentage change in total averaged close to 11 years of growth. On the various quantities of petroleum sources of Co2 emissions for Texas, note that the emission rate for petroleum products non LPG in the period 1986–through 1987, fell by −1.37% only to rise to 4.91% during 1987–1988. In the following years (1988–1989, 1989–1990, 1991–1992, 1993–1994) the discharge rate of petroleum products in the state grew steadily at 1.09%, 1.48%, 3.33% and 2.59%. Conversely, other instances of drops in the emission rates were in the order of −0.75%, −3.56% and −2.18% during the fiscal years 1990–1991, 1992–1993 and 1994–1995. With the exception of minuscule drops of −2.23% to −0.18% in emissions rates for the state of Texas during 1998–1999, 2002–2003, the percentage of change in petroleum products discharged into the atmosphere soared during 1996–2005. During the periods of 1995–1996, 1996–1997, 1997–1998 Texas saw its emission of petroleum products climb to 11.75%, 3.67% and 2.91% respectively. Additional levels of growth in the emission of petroleum pollutants for Texas consists of 3.13%, and 1.15%, .087%, 1.46%, 0.04% that occurred during 2000–2001, 2001–2002, 2003–2004 and 2004–2005 (Table 4).

The emission rate of LPGs which stood at −8.85% in 1986–1987 soared by 11.95% to 3.36% in the subsequent years (1987–1988, 1988–1989) until a visible decline of −17.6% −1.85% during 1989–1990 and 1990–1991. While emission growth rates picked up steam at 11.5% from 1991–1992, in the proceeding years of 1992 and 1993 it dropped to −5.50% only to rebound again to 6.98% in 1993 and 1994. Down the road, the rising levels of emission rates in LPGs were quite visible in 1994– 1995, 1995–1996 and 1997–1998 with 3.44% percentage points, 11.55%, and 40.2%. Notwithstanding the notable emission rate declines of −7.99%, −8.13%, −9.54 to −8.31 in the later years of 1997–1998, 1998–1999, 1999–2000 2004–2005, it is safe to argue that the pace of LPG emission into the atmosphere remained on the rise for greater period of time. Turning to natural gas concentration into the atmosphere, note that it soared moderately at 3.47, 6.68%, 6.03% and 0.11% between 1986–1987, 1987–1988, 1988 through 1989 and 1989–1990. With less than 2% decline rates in 1990–1991, 1991–1992, 1993–1994, natural gas discharge into the atmosphere surged further to 3.86 in 1992– 1993, 4.03% in 1994–1995 and 5.88% in 1995–1996. Just as the remainder of the years represent a mix of declines and increases in the emission rates for natural gas. The temporal profile of change for the combined total of the variables as presented in the table seemed somewhat similar to the other ones herein analyzed (Table 4).

Aside from the miniscule levels of changes in the amount of gas vented and flared during 1986–2000, the growth in the quantity of gas lost during extraction, were quite sizable over the years with periods of intermittent drops as well. During 1986–1987 all through 1987–1988 periods, the amount of gas the study area lost during extraction grew at the rates of 12.5% to 8.54% respectively. In the following years of 1987–1988 and 1988 through 1989, those numbers gradually changed when the amount of gas lost during extraction dropped by −6.47% and −5.11%. Further along the years (1991–1992, 1997–1998, 1999–2000), when some of the biggest levels of declines in the amount of gas lost during extraction stood at −35.7%, −54% and −10.2%, the growth in rate of the amount of gas lost during extraction reached an all time high of 77.5.1% in 1992–1993. The upward trend continued at 21.8% during 1993–1994, 9.86% in 1994–1995, 5.59% in 1996–1997 and 37.4% in 1998–1999. While interstate movement of gas did drop over the years, the upward swing in 6 of the 14 years as shown in the Table are significant enough to impact the quality of the environment in the study area (Table 5).

### Appendix B. Explanation of the Trends in the Analysis and Pollutant numbers

Some of the changes described in the tables 1–5 may be attributed to demographic factors of population growth, policy changes and technological elements as well. In terms of demography, the population of Texas has been on the rise since the years under analysis. Between 1995 through 2006, the population grew from over 18 million to 23 million plus. These increases in population are a primary leading element of change fueling widespread consumption of energy products of oil and natural gas and the quest for more oil and gas wells in the area. In that light, the sequence of gains and declines in these variables point to the pressures mounted by consumer demands and the growing needs of a timing population in a state where per capita consumption of energy products is one of the highest in the entire United States. Additionally, population levels routinely raises people’s use of natural resources and it elevates the amount of pollution and the discharge of large quantities of petroleum sources of CO_2_ and other cancer causing substances into the atmosphere. This is quite critical during those years in which the emission levels stayed on the rise. Conversely, the years of reduced emission of petroleum sources of CO_2_ may be attributed to the enforcement of policy requirements on producers and the recourse to the use of the latest advances in environmentally benign technologies in the sector. The same can be said also be of the sudden drops in the amount of gases lost during extraction in some of the years. All these come at a time when producers and the government are investing heavily in precision technology to ensure better extraction and cost saving measures and conservation of energy.
